# Photocatalytic degradation of antibiotics and antimicrobial and anticancer activities of two-dimensional ZnO nanosheets

**DOI:** 10.1038/s41598-024-59842-6

**Published:** 2024-05-06

**Authors:** Abhik Bhuin, Saranya Udayakumar, Janani Gopalarethinam, Debdyuti Mukherjee, Koyeli Girigoswami, Caroline Ponraj, Sujoy Sarkar

**Affiliations:** 1 Physics Division, School of Advanced Sciences, Vellore Institute of Technology Chennai, Vandalur-Kelambakkam Road, Chennai, Tamil Nadu 600127 India; 2 Medical Bionanotechnology Laboratory, Faculty of Allied Health Sciences, Chettinad Hospital and Research Institute (CHRI), Chettinad Academy of Research and Education (CARE), Chettinad Health City, Kelambakkam, Chennai, 603103 India; 3 Centre for Fuel Cell Technology (CFCT), International Advanced Research Centre for Powder Metallurgy and New Materials (ARCI), IIT-M Research Park, Taramani, Chennai, 600113 India; 4 Chemistry Division, School of Advanced Sciences, Vellore Institute of Technology Chennai, Vandalur-Kelambakkam Road, Chennai, Tamil Nadu 600127 India; 5 Electric Vehicle Incubation, Testing and Research Centre (EVIT-RC), Vellore Institute of Technology Chennai, Vandalur-Kelambakkam Road, Chennai, Tamil Nadu 600127 India

**Keywords:** ZnO nanosheet, Photocatalysis, Antibiotic degradation, Anticancer, Sunlight, Antimicrobial activity, Environmental sciences, Energy science and technology, Materials science, Nanoscience and technology

## Abstract

Active pharmaceutical ingredients have emerged as an environmentally undesirable element because of their widespread exploitation and consequent pollution, which has deleterious effects on living things. In the pursuit of sustainable environmental remediation, biomedical applications, and energy production, there has been a significant focus on two-dimensional materials (2D materials) owing to their unique electrical, optical, and structural properties. Herein, we have synthesized 2D zinc oxide nanosheets (ZnO NSs) using a facile and practicable hydrothermal method and characterized them thoroughly using spectroscopic and microscopic techniques. The 2D nanosheets are used as an efficient photocatalyst for antibiotic (herein, end-user ciprofloxacin (CIP) was used as a model antibiotic) degradation under sunlight. It is observed that ZnO NSs photodegrade ~ 90% of CIP within two hours of sunlight illumination. The molecular mechanism of CIP degradation is proposed based on ex-situ IR analysis. Moreover, the 2D ZNO NSs are used as an antimicrobial agent and exhibit antibacterial qualities against a range of bacterial species, including *Escherichia coli*, *Staphylococcus aureus*, and MIC of the bacteria are found to be 5 μg/l and 10 μg/l, respectively. Despite having the biocompatible nature of ZnO, as-synthesized nanosheets have also shown cytotoxicity against two types of cancer cells, i.e. A549 and A375. Thus, ZnO nanosheets showed a nontoxic nature, which can be exploited as promising alternatives in different biomedical applications.

## Introduction

Technology development in healthcare has positive impacts on the environment and human health, however, improper uses of these advancements cause serious human health issues^1–3^. To address the expanding global concern of antibiotic resistance, the World Health Organization (WHO) has issued several statements and guidelines on the use of antibiotics in healthcare. Antibiotic resistance transpires when bacteria and other microorganisms develop resistance to the particular drugs that are effective in treating the infections^4–7^. This problem leads to the production of antibiotic-resistant DNA and bacteria, which accelerates the spread of antibiotic resistance and poses a threat to human health and the environment^8,9^. Thus, in the twenty-first century, antibiotic contaminants in our water resources are regarded as one of the world's most significant environmental challenges, not only because of environmental damage but also because of the potential impact on human health^10^. According to Murray’s prediction, it has been estimated that if antibiotic resistance continues, the worldwide mortality rate will surpass that of HIV or malaria^2,11^. Antimicrobial resistance is the result of bacteria, fungi, viruses, and parasites that evolve over time and become resistant to drugs, making diseases more difficult to treat and raising the likelihood of disease transmission, fatalities, and death.

Antibiotics can enter the environment through various pathways, such as wastewater discharge, agricultural runoff, pharmaceutical waste^12,13^, aquaculture, etc. Once antibiotics are released into the environment, they can persist for extended periods, potentially affecting ecosystems^14–17^. Efforts are being made to reduce the environmental release of antibiotics, including improved wastewater treatment processes, proper disposal of pharmaceutical waste, or degrading the antibiotics in different ways^18–21^.

Nanotechnology is the field of science that has contributed extensively to the biomedical field including the development of biosensors^22^, biomedical imaging^23,24^, nano formulation of nutraceuticals^25^ as well as wastewater remediation^26,27^. Due to its low cost, high efficiency, and environmental friendliness when it comes to breaking down antibiotics or other pollutants in the presence of sunlight and ambient circumstances, photocatalysis has drawn a lot of interest. In contrast to other promising methods, photocatalysis doesn’t require any additional chemical and eliminates potential contamination with generation of disinfection by-products. Several semiconductor materials, including metal oxides, sulphides C_3_N_4_, BiOX (X = Cl, Br and I), graphene, and transitional metal chalcogenides (TMDs), have been demonstrated effectively in the process of photocatalysis for the degradation of various pollutants, including bacteria^28–34^. Among them, the scientific community has shown considerable interest in ZnO on account of its physicochemical properties, in addition to its cost-effectiveness and natural availability^35–38^. ZnO is well-studied multifunctional semiconductor material with broad application in solar-driven catalysis processes such as water splitting, waste-water treatment, and antibiotic degradations^39–42^. On the other hand, it has unique anticancer and antimicrobial and antifungal properties^29,43–45^ which can be initiated without the presence of sunlight^46^.

Recent studies have shown that researchers have explored ZnO with various morphologies for various applications, including dye degradation, water purification, and anti-bacterial and anti-cancer activities. Verma et al.^47^ report the rod-like shape ZnO NP and their efficiency of 95.9% against Congo Red and a 13 mm, 10 mm zone of inhibition for *E. coli*, and *S. aureus* because of excellent ROS generation capacity and ample Zn^2+^ ions from this semiconducting material. Parthasarathy et al.^48^ explores the ZnO as an alternative to chemotherapeutic human melanoma A375 cells and report that it can cause apoptosis because it makes a lot of ROS, suggesting a synergistic co- relation between semiconductor and their capabilities of photodegradation, anti-bacterial and anti-cancer applications. Babayevska et al.^46^ demonstrated the effect of shapes and sizes of nano- and microparticles of ZnO on cytotoxicity towards normal and cancer cells and antibacterial activity toward different kinds of bacteria. Two- dimensional (2D) materials exhibit superior properties compared to their bulk counterparts, making them highly suitable for wide range applications^49–52^. Typically, these 2D nanomaterials exhibit the generation of electron–hole pairs and subsequent charge separation when subjected to solar irradiation.

In the present study, we have synthesized 2D ZnO NSs using the hydrothermal technique and characterized them using different physiochemical characterization techniques such as XRD, DRS, XPS, SEM, and TEM. The band gap was calculated for these synthesized nanosheets which corroborated with the semiconductor nature of the ZnO nanostructures. We selected ciprofloxacin, a fluoroquinolone antibiotic that treats various bacterial infections, as a model system for our photocatalytic antibiotic studies under natural sunlight. The real antibiotic was degraded successfully using ZnO NSs as a photocatalyst under exposure to sunlight and it was found that ~ 90% of the sample was degraded within two hours. The antimicrobial activities of Gram-negative and Gram-positive bacteria such as *E. coli* and *S. aurous,* respectively were investigated using different doses of as-synthesized ZnO NSs. Apart from these, synthesized catalyst has also shown cytotoxicity against two types of cancer cells, i.e. A549 and A375 and their biocompatibility was investigated via in vitro cell culture.

## Results and discussion

### Structural and morphological properties

**Figure 1:**
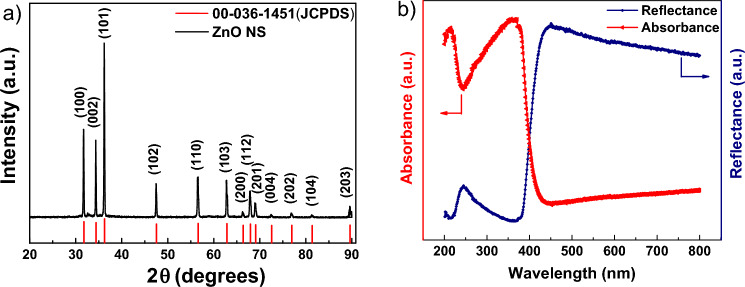
(**a**) XRD of as-synthesized ZnO nanosheets (black lines) along with JCPDS reference (red lines); (**b**) Diffuse reflectance spectra of ZnO nanosheets, where red curve for absorbance and blue curve for reflectance.

X-ray diffraction technique was employed to characterize the as-synthesized ZnO (1:1 molar ratio) crystal structures and phase compositions. The diffraction peaks (Fig. 1a) at 31.72, 34.36, 36.18, 47.46, 56.52, 62.72, and 66.22, which correspond to the (100), (002), (101), (102), (110), (103), and (200) crystal planes, respectively, revealed the successful preparation of ZnO with a hexagonal wurtzite structure. No additional peak can be observed in the XRD curve of the as-synthesized materials, indicating a high purity of the product. The acquired characteristic peaks for the ZnO were well matched with the crystal planes of hexagonal ZnO (JCPDS card no. #36-1451), suggesting about the sample purity and high crystallinity.

The crystallite size was calculated using Debye–Scherrer equation (Eq. 1) for the most intense peak at (101) plane:

1 $$D=\frac{k\lambda }{\beta Cos\theta }$$

where D = crystallite size; λ = wavelength of X-ray; k = shape factor (0.89); β = FWHM (full width at half maximum of (101) plane) (in radians); θ = angle of diffraction (in radians);

The estimated size of the ZnO nanosheets was found to be ~ 56 nm from the above equation.

The optical property of ZnO NSs was determined using UV–vis diffuse reflectance spectroscopy in the presence of BaSO_4_ crystal in a wavelength range of 200–800 nm. The UV–vis absorbance and corresponding diffuse reflectance spectra of ZnO NSs are shown in Fig. 1b, which exhibits a typical absorption behaviour of the wide band gap semiconductor and found the absorbance band edge of ZnO NSs at 430 nm.

The band gap of the ZnO NSs can be calculated using the Kubelka–Munk function (F(R), according to P. Kubelka and F. Munk's 1931 theory^53^. The recorded reflectance spectra can be converted to the matching absorption spectra by applying the Kubelka–Munk function (F(R∞) and using the following equation (Eq. 2).

2 $$(F(R_{\infty } )h\nu )^{\gamma } = A(h\nu - E_{g} )$$

where h = Planck’s Constant, ν = frequency of the photon, A = Proportionality Constant; *E*_g_ = Band Gap of the material; γ = nature of electron transition (= 2 for direct allowed transitions). The band gap of the as-synthesized ZnO NSs is found to be 3.2 eV.

**Figure 2:**
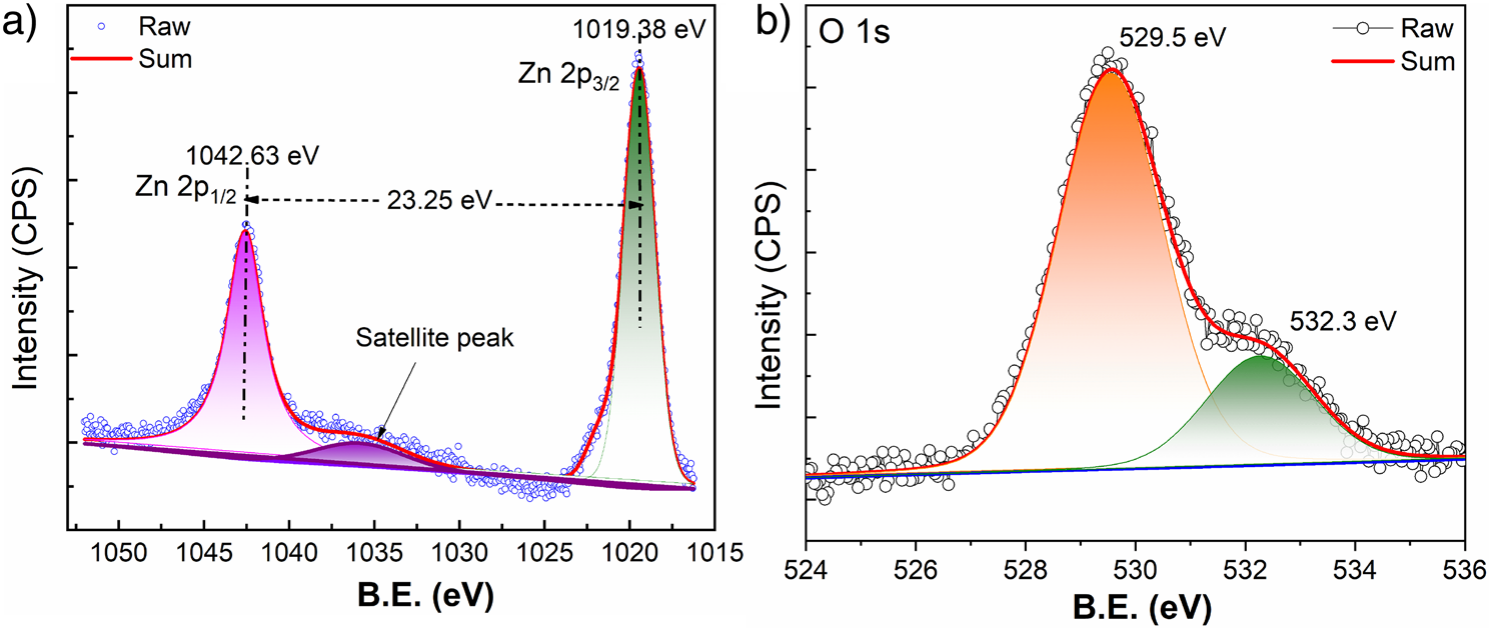
(**a**) High resolution deconvoluted Zn 2p and (**b**) O 1s of as-synthesized ZnO NSs.

The X-ray photoelectron spectroscopy (XPS) is a powerful tool for investigating the complexities of electronic structures of solids. The ZnO nanosheets were analysed by XPS and displayed in Fig. 2. It shows the typical XPS wide survey spectra (Fig. S1) of ZnO nanosheets where Zn, O, and C peaks were detected which establishes that Zn and O are the main constituents and prepared nanosheets are absolutely free from extraneous impurity. The detected carbon is related to the reference carbon and all binding energies are calibrated considering the C 1s emission centred at 284.5 eV as a reference^54^.

**Figure 3:**
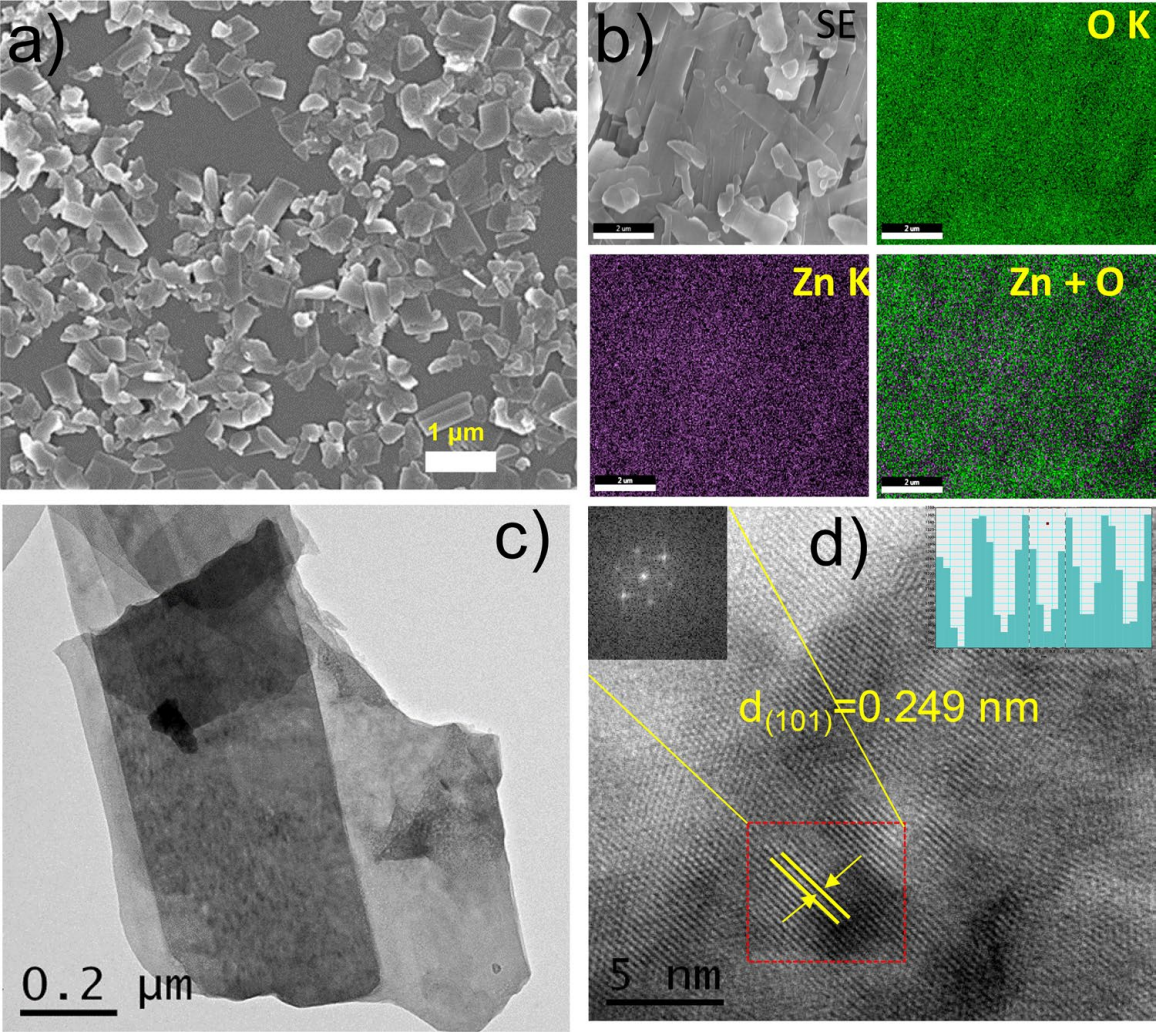
FESEM image of ZnO NSs; (**a**) lower magnification; (**b**) elemental mapping of Zn and O along with SE image; (**c**) Bright field transmission electron microscopic image of ZnO NSs and (**d**) high resolution TEM image with d-spacing of 0.249 nm corresponding to (101) reflection (zoomed d-spacing is shown in inset) along with Fourier transform of thin and isolated portion of the sheet marks with red square region. The lattice fringe region of the crystal line profile with computed length scale is shown in right inset.

Figure 2 demonstrates the deconvoluted raw data of high-resolution XPS spectra of the Zn 2p region (a) and O 1s core-level (b) of ZnO nanosheets. It is found that the Zn 2p core-level of ZnO NSs has two deconvoluted peaks located at about 1044.5 and 1021.5 eV attributed to Zn 2p_1/2_ and Zn 2p_3/2_, respectively. This result implies that the chemical valence of Zn at the surface of nanosheets is + 2 oxidation state. The binding energy difference between the Zn2p_1/2_ and Zn 2p_3/2_ is found to be 23 eV due to the spin–orbit splitting. Figure 3b displays core-level O1s region of ZnO nanosheets where experimental data was deconvoluted into two Gaussians peaks, positioned at the lower binding energy of 529.5 eV that is assigned to O^2−^ ion in the Zn–O bonding of the wurtzite structure of ZnO. The second peak located at 532.6 eV is related to the hydroxyl group absorbed onto the surface of the nanosheets^55^.

The ZnO NSs were analyzed further by FESEM technique to observe their morphology and structure. The current study revealed that as-synthesized ZnO NSs formed a two-dimensional nanosheet structure and depicted in Fig. 3a. The average nanoparticle size was found to be 240 nm (Supporting information, Fig S2). The energy dispersive spectroscopy (EDS) further confirms the presence of Zn and O (Supporting information, Fig. S3), and the atomic % ratio of O and Zn was found to be 1:1.16. Furthermore, Fig. 3b depicts the elemental mapping of the ZnO NSs, demonstrating the homogeneous distribution of Zn and O elements across the ZnO NSs as shown in the SE image. The even colour distribution Zn and O confirms the proper synthesis of ZnO NSs.

Further, Transmission Electron Microscopy (TEM) provides a more detailed and higher-resolution image of ZnO. The bright field TEM image (Fig. 3c) of the ZnO NSs clearly reveals the sheets-like structure. The high-resolution TEM (HRTEM) image (Fig. 3d) indicates the crystalline phase with a d-spacing of 0.239 nm corresponding to the (101) plane. This was determined from the lattice fringe region of the crystalline profile with a computed length scale. The fast Fourier transform (FFT) of the HRTEM image shown in the inset of Fig. 3d confirms the formation of ZnO in a hexagonal wurtzite structure. The lattice parameters calculated from the FFT pattern match very well with that of ZnO. For ZnO NSs, it was obtained a specific surface area of 5.4 m^2^ g^−1^ by the BET method.

### Antibiotic degradation

Prior to antibiotic degradation studies, the photocatalytic activity of ZnO NSs was investigated by photodegradation of methylene blue (MB) aqueous solution in direct sunlight. The solutions were held in the dark for half an hour before irradiation to reach photocatalyst-pollutant adsorption–desorption equilibrium. After that, the sample was exposed to sunlight and measured the sample at different irradiation time (Fig. S4). In order to explore the activity of the ZnO NSs for MB degradation, different factors like irradiation time, material dosage, dye concentration, different molar ratio, and pH of the solution were investigated and displayed in the Fig. S5. Moreover, the catalyst’s reusability was also examined and depicted in Fig. S6. This experiment motivates us to investigate the photodegradation studies of another harmful organic molecules such as unused antibiotic using the as-synthesized ZnO NSs.

**Figure 4:**
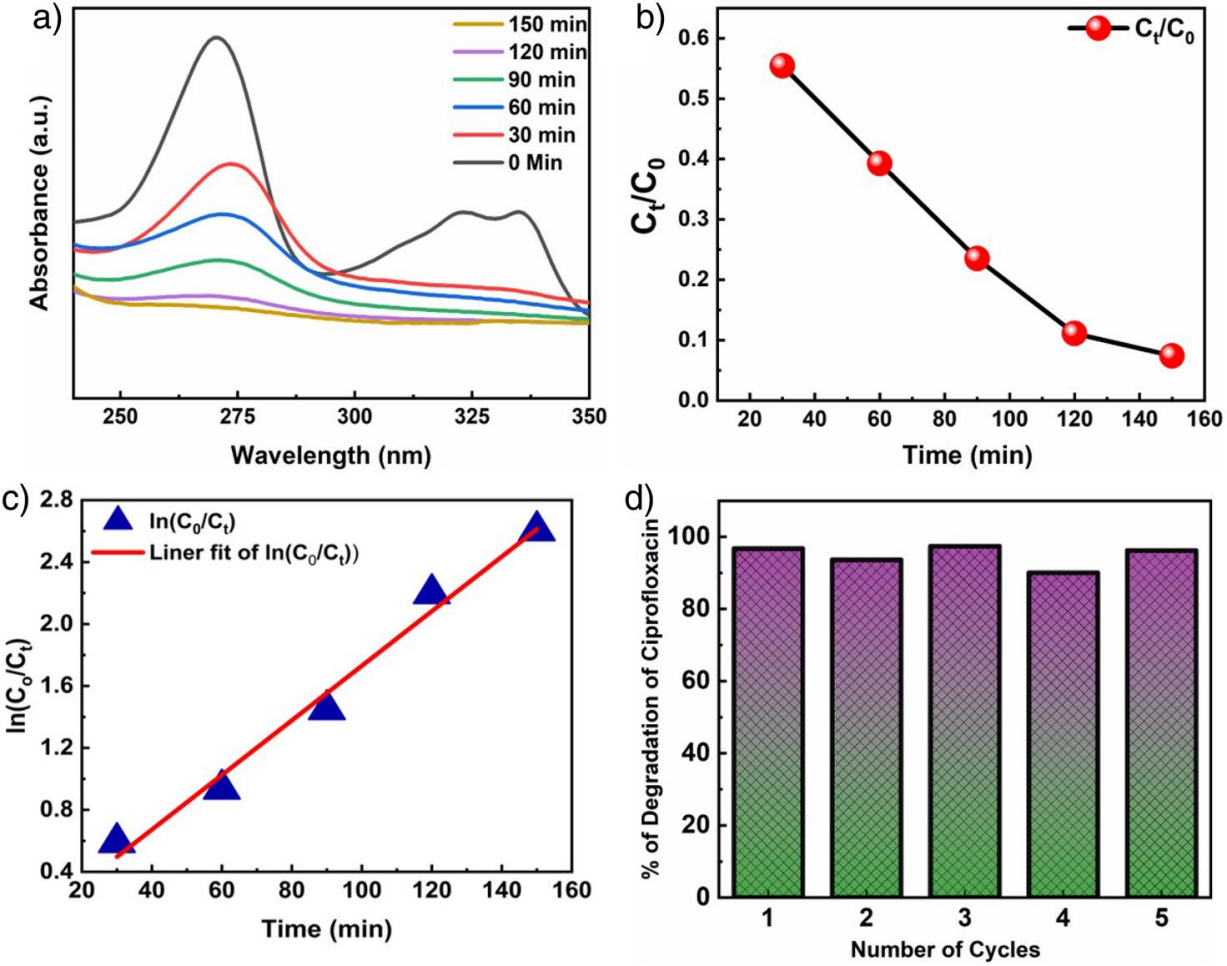
(**a**) Time-dependent UV − vis spectra of photocatalytic degradation of liquid-phase CIP under direct sunlight of ZnO NSs. (**b**) Fist-order plot for the photo-induced oxidation of (CIP). (**c**) Time-dependent liquid-phase photocatalytic degradation of CIP. (**d**) Reusability study of ZnO NSs in CIP degradation for five consecutive cycles.

The photocatalytic degradation studies of an end-user antibiotic (here, Ciprofloxacin (CIP) tablets from local medicine store) was investigated under natural sunlight. Figure 4a shows the degradation profile of CIP by ZnO NSs under direct sunlight irradiation. The absorption maximum of the drug (10^–5^ M) was reduced to 60% after 60 min exposure of the direct sunlight and almost 90% of the CIP degraded within 120 min exposure of the sunlight. The slight red shift of the degraded peaks is well in agreement with the reported literature^41,56^. The intensity of the peak at 271 nm was measured to track the development of the photocatalytic degradation of CIP. The kinetics of the photodegradation of CIP was determined using Eq. 3

3 $$\ln \left( {C_{0} /C_{t} } \right) = kt$$

where “C_0_” is the initial concentration of the CIP solution, “C_t_” is the concentration of CIP at a time “t”, and k is the first-order rate constant, which was calculated from the slope of the plot. The percentage of photocatalytic degradation (PD) was calculated by the formula as given by Eq. 4^56^.

4 $$PD\,\left( \% \right) = [C_{t} /C_{0} ] \times 100$$

Figure 4b represents the photocatalytic degradation of CIP using the concentration of CIP as a function of degradation time. A strong linear correlation can be observed between ln(C_0_/C_t_) and the degradation time (Fig. 4c). A first-order kinetic model fits to explain the photocatalytic degradation of CIP and the data exhibited a strong fit with an R^2^ value close to 0.99, implying that the photodegradation mechanism follows a pseudo-first-order kinetic model. The rate constant and half-life of the degradation are found to be 0.0174 min^−1^ and 40 min, respectively. Almost linear nature of the curve till 150 min of degradation studies emphasises on the strong performance of ZnO NSs against CIP. The summary of the photodegradation of CIP is tabulated in Table 1.

**Table 1:** Summary of photodegradation of CIP using ZnO NSs under natural sunlight.

Sample name	Amount of sample used (mg/ml)	% of degradation/degradation efficiency	Rate const. (min^−1^)	Half-life (min)	R^2^
ZnO NSs	0.5	92.6% in 150 min	0.0174	40	0.99

Figure 4d represents reusability of the photocatalyst and the photocatalyst was used for five runs under visible light irradiation. It was executed to assess the CIP degradation efficiency and reusability of as synthesized ZnO NS. It is noteworthy that the ZnO NSs continued the high CIP degradation activity in all 5 rounds exhibiting good photostability under sunlight irradiation and its photocatalytic efficiency only reduces by 5% after five repeated cycles. Further, the photocatalyst was investigated using XRD (Fig. S6) for structural integrity and no change was observed. This indicates that the ZnO NSs maintained their structural stability and photocatalytic stability throughout the process.

**Scheme 1:**
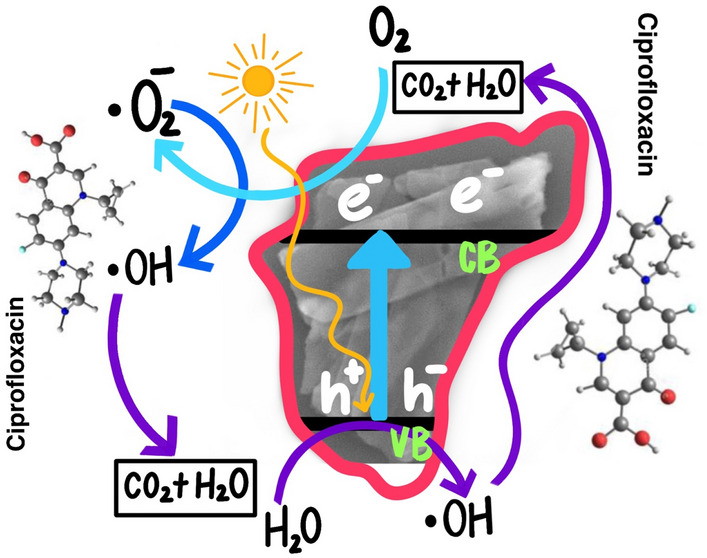
Schematic representation of photocatalytic degradation of ciprofloxacin using ZnO NSs.

To understand the CIP degradation under sunlight using ZnO NSs, a plausible mechanism is proposed based on the literature report^57^. At first, ZnO NSs photocatalysts were exposed to sunlight to excite electrons from the valence band to the conduction band. According to the equations and Scheme 1 below, the electron–hole pair is produced when an electron is excited from the valence band to the conduction band. When water molecules reacted with the hole (h^+^), hydrogen ion (H^+^), hydrogen peroxides(H_2_O_2_), and hydroxyl free radicals (^·^OH) were produced. As shown in Scheme 1, the H_2_O_2_ produced in situ was fragmented into two ^·^OH radicals, which were in charge of the majority of the ciprofloxacin degradation. The conduction band electrons (e) helped to produce superoxide free radicals (^·^O_2_^−^). They were joined to create the H_2_O_2_ molecule because reactive oxygen species (ROS) are so highly reactive. As the H_2_O_2_ molecule broke apart, the ciprofloxacin was degraded. The corresponding chemical equations as described in Eqs. 5–10

5 $${\text{ZnO}} + {\text{h}}\vartheta \to {\text{h}}^{ + } ({\text{VB}}) + {\text{e}}^{ - } ({\text{CB}})$$

6 $$h^{ + } {\text{(VB)}} + {\text{H}}_{2} {\text{O}} \to {\text{H}}^{ + } + \,^{ \cdot } {\text{OH}}$$

7 $${\text{e}}^{ - } {\text{(CB)}} + {\text{O}}_{2} \to {\text{CB}} + \,^{ \cdot } {\text{O}}_{2}^{ - }$$

8 $$\,^{ \cdot } {\text{O}}_{2}^{ - } + 2{\text{H}}^{ + } + 2^{ \cdot } {\text{OH}} \to {\text{O}}_{2} + 2{\text{H}}_{2} {\text{O}}_{2}$$

9 $$2{\text{H}}_{2} {\text{O}}_{2} \to 4\,^{ \cdot } {\text{OH}}$$

10 $$\,^{ \cdot } {\text{O}}_{2}^{ - } + \,^{ \cdot } {\text{OH}} + {\text{CIP}} \to {\text{H}}_{2} {\text{O}} + {\text{CO}}_{2}$$

To understand the molecular mechanism of CIP degradation under sunlight using ZnO NSs, a plausible mechanism is proposed. According to the literature^42^, there are four different mechanisms predicted for photocatalytic oxidation of CIP, such as cleavage of the piperazine ring, defluorination, decarboxylation, and oxidation of the cyclopropyl group. To understand the photodegradation, CIP samples at different intervals, such as 0 min, 30 min, and 120 min were collected and examined under InfraRed (IR) spectroscopy (Fig. S7a) and found that the peak at 1633 cm^−1^ (assigned to –C=O stretching vibration) is increased (Fig. S7b) which reveals the increasing of –C=O functional groups in the degraded products^58^. Moreover, the peak at 1275 cm^-1^ (Fig. S7c; assigned to C-N stretching vibration) completely disappeared in the CIP irradiated with 120 min sunlight. It suggests the cleavage of the piperazine moiety and the formation of Intermediate 1 (I), which subsequently degraded and formed CO_2_ and H_2_O along with some inorganic ions. From the IR spectra, we can speculate that CIP photodegraded through the cleavage of the piperazine ring path. The proposed plausible mechanism is shown in Scheme 1.

To understand the CIP degradation further, the catalyst dosage, CIP concentration and pH of the solution were varied and displayed in the Fig. S8.

### Antimicrobial activity

**Figure 5:**
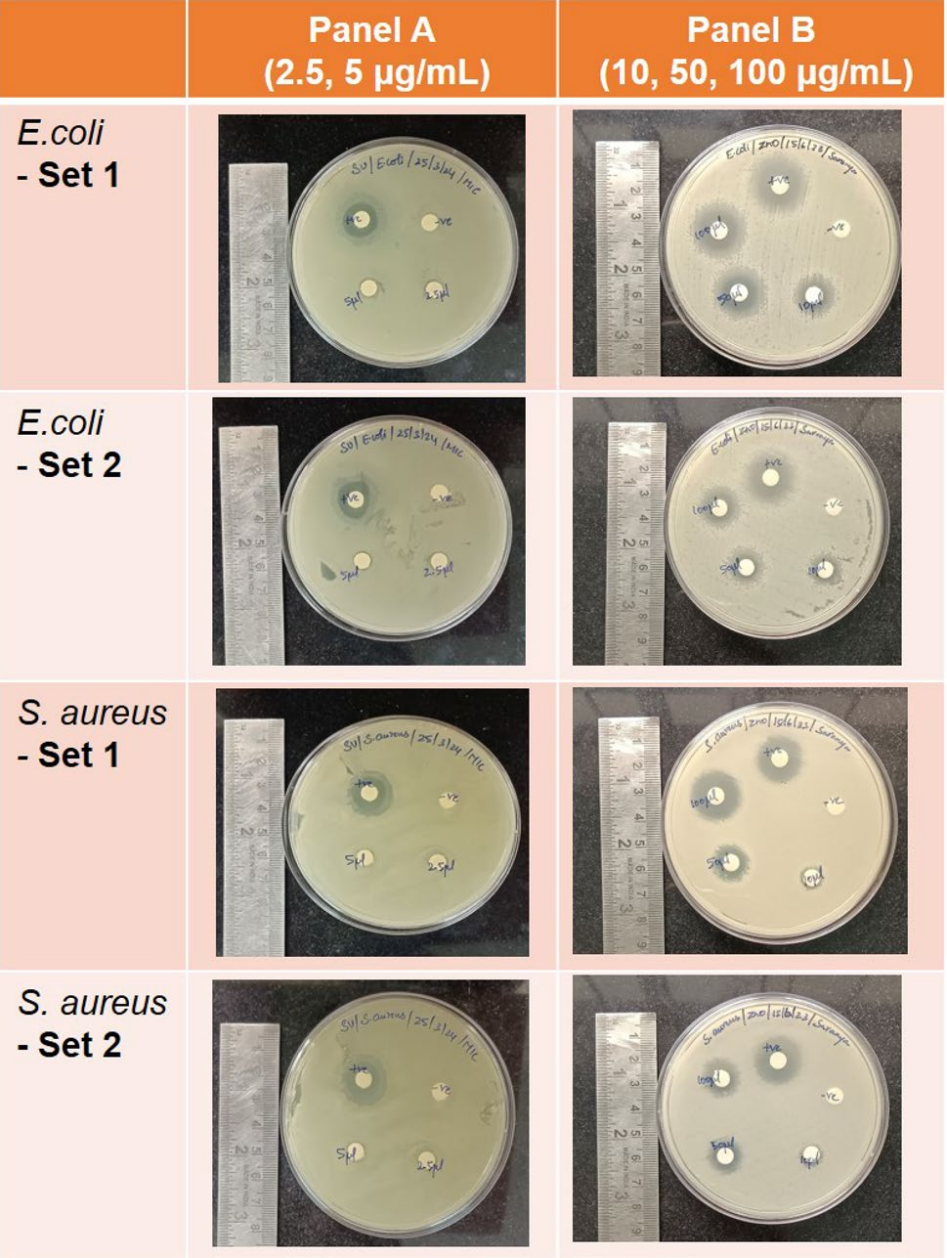
The zone of inhibition for ZnO NSs for *E. coli* and *S. aureus* at different concentrations (0, 2.5, 5, 10, 50, and 100 μg/ml). The diameter of clear zone is shown in Table 2 and minimum inhibitory concentration (MIC) was observed to be 5 μg/mL for *E. coli* and 10 μg/mL for *S. aureus*.

**Figure 6:**
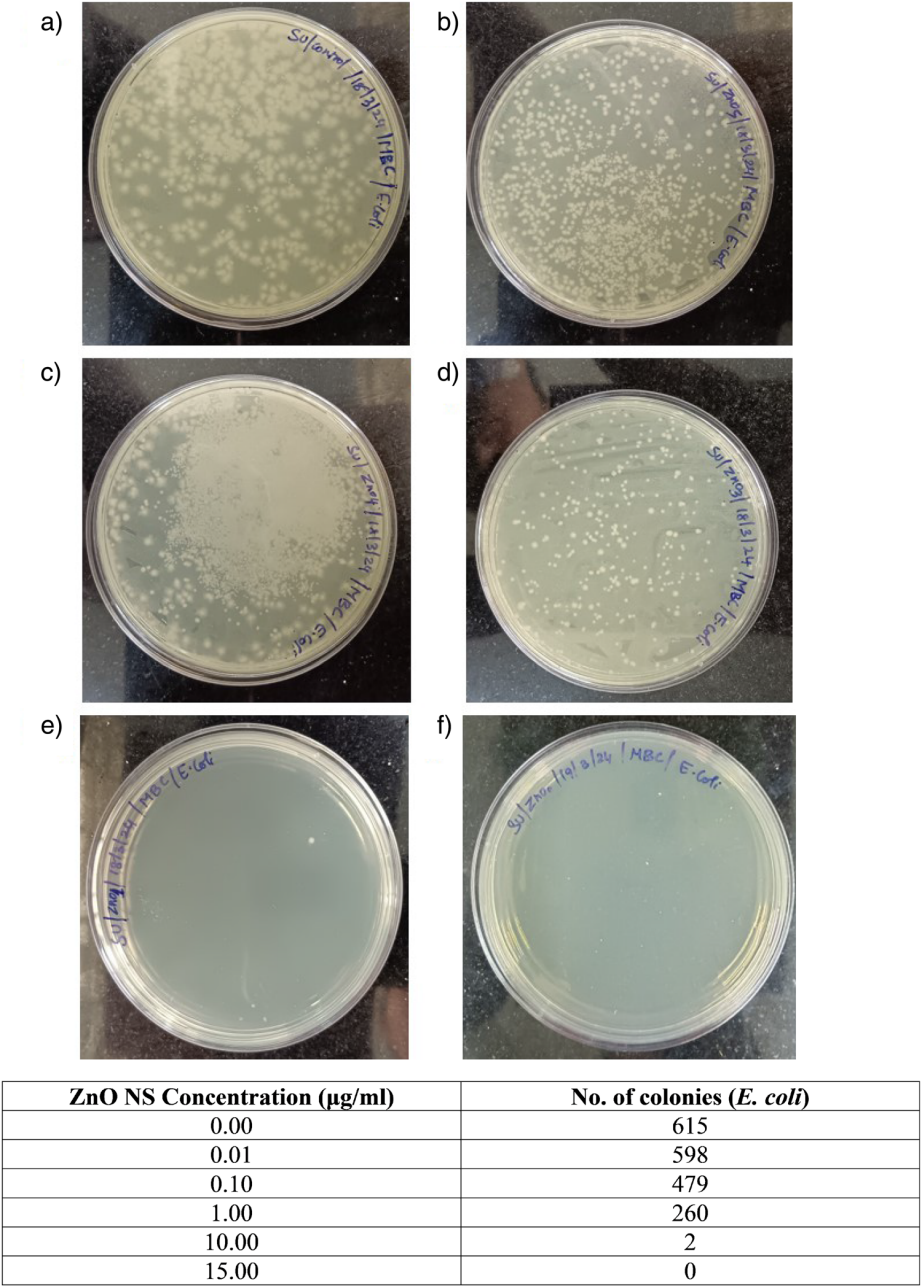
The bacterial colonies were counted for the different concentrations of ZnO NSs treated *E. coli* using spread plate technique. (**a**) untreated control, (**b**) 0.01 μg/mL ZnO, (**c**) 0.1 μg/mL ZnO, (**d**) 1.0 μg/mL ZnO, (**e**) 10 μg/mL ZnO, (**f**) 15 μg/mL ZnO, (**g**) table showing the number of colonies counted for the different plates (untreated and treated with ZnO).

**Figure 7:**
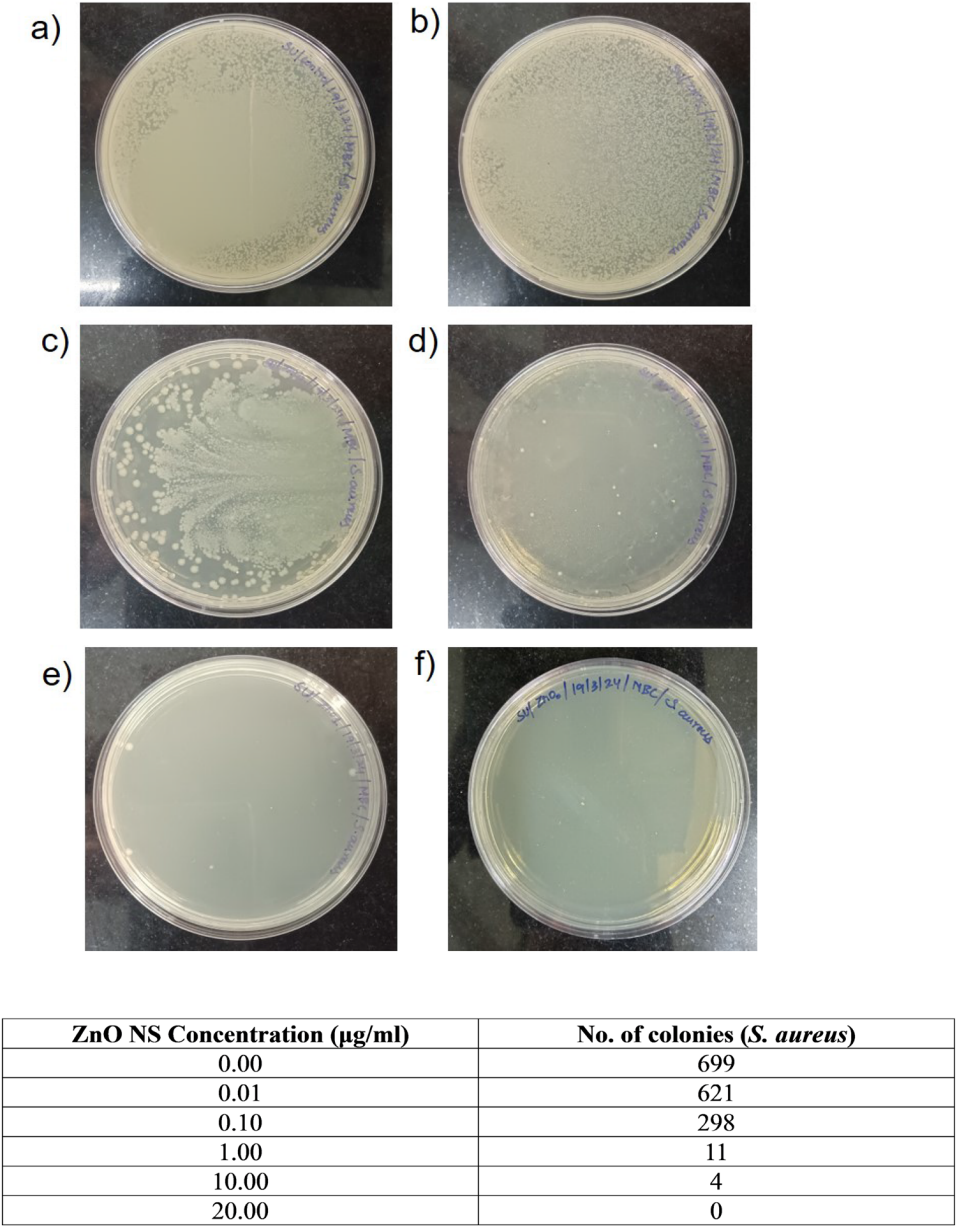
The bacterial colonies were counted for the different concentrations of ZnO NSs treated *S. aureus* using spread plate technique. (**a**) untreated control, (**b**) 0.01 μg/mL ZnO, (**c**) 0.1 μg/mL ZnO, (**d**) 1.0 μg/mL ZnO, (**e**) 10 μg/mL ZnO, (**f**) 20 μg/mL ZnO, (**g**) table showing the number of colonies counted for the different plates (untreated and treated with ZnO).

The microbial susceptibility of ZnO-NSs varies depending on the specific bacteria and the quantities of the ZnO-NSs. The antibacterial activity of ZnO NSs against *E. coli* (Gram-negative) and *S. aureus* (Gram-positive) was investigated, and the results are presented in Fig. 5. The Bonferroni test revealed that the growth of *E. coli* and *S. aureus* bacteria was observed in a dose-dependent manner at higher concentrations (10 μl, 50 μl, 100 μl from a stock of 10 mg/ml), which is consistent with the findings reported by Galindo et al.^35^. A zone of inhibition is formed for measured values of 10 μg/mL, 50 μg/mL, and 100 μg/mL, but for lower concentrations (2.5 and 5 μg/mL), there was no significant inhibition for *S. aureus*, whereas for *E. coli*, a zone of inhibition was visible at 5 μg/mL. This experiment shows that the MIC of *E. coli* is 5 μg/mL and *S. aureus* is 10 μg/mL. The antibacterial efficacy of ZnO-NSs against various microorganisms was evaluated using the disc diffusion method where disinfectant-sensitive microorganisms have large inhibition diameters, while resistant pathogens have shorter or no inhibition diameters. Antibacterial activity (MIC) of ZnO NSs shows 18 mm, and 21 mm of inhibition zone for *E. coli,* while for *S. aureus*, ZnO NSs exhibit 20 mm and 19 mm of inhibition zone as displayed in Table 2. It is noteworthy that the inhibition zone diameter at a higher dose (i.e., 100 μg/mL) is comparable to the standard antibiotic solution for both Gram-positive and Gram-negative bacteria. In the case of S. aureus, the zone of inhibition was higher than the standard antibiotic solution at 100 μl (from 10 mg/mL) showing the superior capacity of the ZnO NSs towards antimicrobial activity for Gram-positive bacteria. A step further, we explored the minimum bactericidal concentration of the synthesized ZnO NSs. The results are shown in Fig. 6 for *E. coli* and Fig. 7 for *S. aureus*. From the results it was noted that at concentration of 10 μg/mL there are only two colonies present for *E. coli*, and at 15 μg/mL no colonies were present. This showed that for *E. coli*, the MBC was 15 μg/mL. On the other hand, for *S. aureus* (Fig. 7), it was observed that at 10 μg/mL, there were 4 colonies present but at 20 μg/mL, no growth was observed. This concluded that the MBC for *S. aureus* was 20 μg/mL. It is found in the literature^35^ that the antibacterial effect is allied to the size of the nanoparticle^59^ too. Nevertheless, according to research findings, the detrimental effect of NS on microorganisms increases with the declining size of nanoparticles^60,61^. From the SEM images (Fig. S9) of bare *E. coli* and *E. coli* treated with 10 μg/ml of ZnO NSs, it can be clearly seen that the bacteria have a typical rod- shaped morphology (Fig. S9a), with an intact cell wall. On the other hand, after treatment with ZnO NSs (Fig. S9b), there was aggregation of nanoparticles above the bacteria. The cell wall is also ruptured giving an irregular shape of the coliform bacteria. In case of *S. aureus*, typical coccus shaped is observed in the Fig. S9c. After treatment with 10 μg/ml of ZnO NSs, it was found that the cell wall was also ruptured causing it to get killed. The ruptured cell wall of *S. aureus* can be observed in Fig. S9d. This shows that our synthesized ZnO nanosheets were capable of killing both Gram positive as well as Gram negative bacteria as visible through SEM images.

**Table 2:** The zone of inhibition was measured for *E. coli* and *S. aureus* after treatment with different concentrations of ZnO nanosheets.

Sample	Culture	Concentration µg/mL	Zone of inhibition (cm)		
			Plate 1	Plate 2	Average ± S.D. (cm)
ZnO NSs	*E. coli*	0 (negative control)	0.7	0.8	0.75 ± 0.07
		2.5	0.8	0.8	0.8 ± 0.00
		5.0	0.9	1.0	0.95 ± 0.07
		10	1.4	1.7	1.55 ± 0.21
		50	1.6	1.9	1.75 ± 0.21
		100	1.8	2.1	1.95 ± 0.21
		Positive control (antibiotic solution)	2.1	2.1	2.1 ± 0.00
	*S. aureus*	0 (negative control)	0.7	0.8	0.75 ± 0.07
		2.5	0.7	0.8	0.75 ± 0.07
		5.0	0.8	0.8	0.8 ± 0.00
		10	0.9	0.8	0.85** ± **0.07
		50	1.5	1.5	1.5** ± **0.00
		100	2	1.9	1.95** ± **0.07
		Positive control (antibiotic solution)	2	1.7	1.85** ± **0.21

ZnO NSs have antibacterial properties, although their exact mechanism of action is still not entirely understood. Despite this, numerous investigations have demonstrated that ZnO NSs has an antimicrobial effect. Potential antibacterial activity against human infections is also provided by ZnONSs. Recently, it was shown that ZnO nanoparticles exhibit antibacterial action against skin-specific bacteria which was made feasible only by their size and morphology, which in turn, is directly proportional to the amount of the dose used for inhibition. ZnO NSs with antibiotic coatings is efficient against bacteria. Both the release of ions, during antibacterial activity and the release of Zn^2+^ ions in aqueous solution by ZnO NSs boosted the effectiveness of the antimicrobial treatment. Both Gram-positive and Gram-negative bacteria have negatively charged cell membranes due to the existence of a negative charge. Before entering the bacterium, zinc ions first establish contact with the bacterial cell membrane's outer surface to cause mechanical damage^62,63^. The generation of ROS (Superoxide ions (O_2_^−^), hydroxyl ions (OH^−^), and hydrogen peroxide (H_2_O_2_)) and zinc ions (Zn^2+^) are two possible mechanisms for how ZnO NSs exert their antibacterial effects. Apart from these ZnO NS has capabilities for enzyme inactivation, interfering with DNA replication, protein denaturation, ribosomal destabilization, disrupting cell walls and membrane, and any combination of all together, which possibly also contribute to the antibacterial properties^64^.

To understand the mechanism behind the antibacterial effect could be related to its capability to mechanically damage the cell membrane via the production of reactive oxidative substances such as OH^-^, H_2_O_2,_ etc. which depends on the active surface area of the materials. It could be possible that the amount of releasing oxidative species is more for nanosheet morphology, which in turn, is directly proportional to the amount of the dose used for inhibition.

### Biocompatibility and anticancer activity

**Figure 8:**
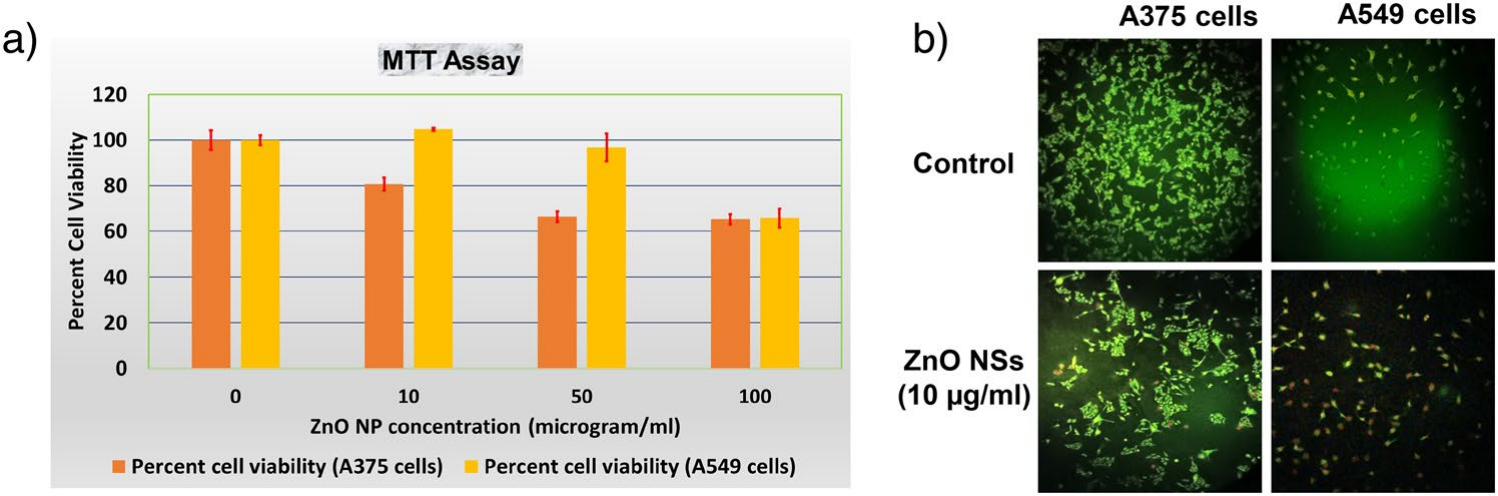
(**a**) The percentage of cell viability of A549 and A375 cells after treatment with different doses of ZnO NSs (5 μg/ml 10 μg/ml, 50 μg/ml, 100 μg/ml) using MTT assay. (**b**) Live dead assay for A549 and A375 cells after treatment with different doses of ZnO NSs (10 μg/ml).

It is well known that to apply any nanostructure to the environment, its biocompatibility must be monitored to avoid any detrimental effects. To assess the biocompatibility, we have done an MTT assay using two types of cancer cell lines A549 and A375 where human lung adenocarcinoma cell line A549 commonly serves as a model for lung cancer research and researchers often use A375, a human malignant melanoma cell line, as a model for melanoma research. Our nanostructure could inhibit the proliferation of skin cancer cells in a dose-dependent manner, whereas the lung cancer cells were killed significantly up to a dose of 50 μg/ml (Fig. 8a). At a 100 μg/ml dose, both cancer cells were killed, showing a viability of nearly 65%. These data were supported by the results of a live dead assay, where we could observe that at a 10 μg/ml dose, the ZnO nanosheets could elicit a greater number of dead cells in A375 cells compared to A549 cells (Fig. 8b). Overall, the experimental data suggested that the synthesized ZnO nanosheets exhibit anticancer activity.

The mechanism behind cell death was explained in the literature^65,66^ as ROS along with apoptosis and necrosis, are the reasons behind cell rupture. However, cell membrane rupture^67^ during the migration of ZnO nanosheets within the cells could be another reason behind the anticancer activities of the materials. ZnO is known to elicit anticancer effects. ROS generation and lipid peroxidation in the cell membrane as well as the membranes of the organelles, when ZnO NSs interact with these components can be a speculated as possible mechanisms of cancer cell killing. In addition to free radical reactions, ZnO NSs cause ROS production by interacting with mitochondrial parts, turning on growth factors, and turning on NAD(P)H oxidases (NO_X_). Nanoparticles induce cellular ROS generation in which mitochondria play a crucial role. A previous study demonstrated that NPs are capable of depolarizing mitochondrial membranes and interfering with the electron-transport chain by activating NADPH-related enzymes^68^. Through electron transfer from respiratory carriers to O_2_, nanoparticles can block mitochondrial electron transport chains, resulting in an increase in the cellular levels of O_2_^·–^ (superoxide radical).

On the other hand, it is known that cancer cells possess a leaky vasculature, which enables the retention of nanoparticles inside them due to the enhanced permeability and retention (EPR) effect. Thus, compared to normal cells, the membrane pore size of the cancer cells is larger, which allows a higher number of nanoparticles, to accumulate inside the cells and elicit the response. The optimum size of the ZnO NSs synthesized in this study could allow these nanostructures to penetrate cancer cells in high amounts and cause cell killing. Our study could conclude that at a higher dose of 100 μg/ml, both skin cancer as well as lung cancer cell lines exhibited high cell death, but for the skin cancer cell line, even a low dose of ZnO NSs (10 μg/ml), was sufficient to lead these cells to die. The obtained experimental results are compared with different ZnO nanostructures and tabulated in Table S1.

## Conclusion

In summary, we have prepared optimized, unique morphology-based 2D ZnO nanosheets through the one-pot hydrothermal method for high photocatalytic activity and ROS generation. The high-purity hexagonal structure was confirmed by XRD, and the band gap was 3.2 eV by DRS. FESEM and TEM confirm the unique 2D morphology, with an average particle size of 240 nm and structure, respectively. EDS and XPS confirm the molecular composition and relative distribution. In multifunction applications, the synthesized material shows strong and competitive results. Harnessing the solar irradiation, ZnO NS was capable of showing degradation even at a 5 × 10^–5^ M concentration and for a 10^-5^ M, ~ 90% degradation in a mere 2 h exposure. The ZnO NSs continued the high CIP degradation activity in all 5 cycles, exhibiting good photostability and reusability under sunlight. At a high concentration of 100 μg/mL, ZnO NS has the same-sized inhibition zone as the standard antibiotic for both Gram-positive and Gram-negative bacteria. Notably, the inhibition zone for *S. aureus* was larger than that of the standard antibiotic at 100 μL from a 10 mg/mL solution. This shows that ZnO NS is more effective at killing Gram-positive bacteria than the standard antibiotic. At a concentration of 10 μg/ml, ZnO NS caused more dead cells in the A375 cell line than in the A549 cell line. This shows that it can stop the growth of skin cancer cells as the concentration increases. While lung cancer cells exhibited significant mortality at concentrations up to 50 μg/ml, at a concentration of 100 μg/ml, the viability of both types of cancer cells was reduced to approximately 65% despite being biocompatible. Potential applications for the developed ZnO NS include the degradation of other fluoroquinolones and antibiotics, as well as other organic contaminants in water that contribute to antibiotic resistance, and the use of sunlight to create a biocompatible and sustainable environment. The prepared 2D ZnO NSs photocatalyst will open up different applications in the fields of cancer diagnostics and therapy, catalysis, photocatalytic water splitting, and water treatment.

## Experimental

### Materials and chemicals

Hexamethylenetetramine (C_6_H_12_N_4,_ 99.5%), zinc nitrate hexahydrate (Zn(NO_3_)_2_.6H_2_O, 99%), and ethanol was obtained from SRL, India. 250 mg CIPZOX (Ciprofloxacin) was obtained from a local pharmaceutical store. The chemicals used in this study were used as a received condition without further purification. The National Center for Cell Science (NCCS) in Pune was able to provide us with human alveolar basal epithelial cells from A549 adenocarcinoma and A375 human melanoma. HiMedia, India, provided Dulbecco's Modified Eagle's Medium (DMEM) and antibiotic solution (penicillin and streptomycin). The Fetal bovine serum (FBS) was purchased from Gibco, USA. Other chemicals such as 3-(4,5-dimethylthiazol-2-yl)-2,5-diphenyltetrazolium bromide, nutrient agar, Luria–Bertani broth, sterile disc (6 mm), ethidium bromide, and acridine orange were supplied by Hi-Media, India. All the chemicals used were AR grade, cell culture tested with purity of 99–100%.

### Preparation of ZnO nanosheet

ZnO nanosheets were synthesized according to Sun et al.^69^ with small modifications. In a typical synthesis, 1:1, molar ratio of zinc nitrate hexahydrate and hexamethylenetetramine (Hexamine) were added to 150 mL of deionized water while being stirred for 5 min in a subsequent manner. A suspended solution was formed. A Teflon-lined autoclave with the suspension inside was used to maintain the hydrothermal reaction at 100 $^\circ$ C for 12 h. The precipitate was repeatedly cleaned with ethanol and deionized water. The obtained ZnO nanosheet (ZnO NSs) was then dried overnight at 100 $^\circ$ C in a normal oven. Similar method was employed for synthesizing ZnO nanostructures with different molar ratio (1:0.5 and 1:1.5) of Zn-precursor and Hexamine and characterized with XRD and SEM (Fig. S10).

### Characterization

X-ray diffraction with a powder diffractometer (Rigaku Smart Lab 3 kW XRD System) and monochromatic Cu-Kα radiation (λ = 1.5418 Å) at a scan rate of 5°/min for 2θ (10°–80°) was applied to analyse structural and crystalline characteristics of the material. FE-SEM (Zeiss, Germany) and HR-TEM (JEOL JEM 2100F) were utilized to analyze interlayer, spacing morphology, and particle size. Using a UV–Vis spectrophotometer (Thermo Scientific, EV300 and Perkin Elmer UV/VIS spectrometer Lambda 35) in the range of 200–900 nm, optical properties were investigated. X-ray Photoelectron Spectroscopy (XPS, Omicron Nano Technology, UK) was used to perform the core level information of the materials. Ex-situ FTIR was performed in ATR mode (Shimadzu, USA).

### Antibiotic degradation

In the antibiotic degradation, 50 mg of photocatalyst were dispersed in 100 mL of CIP solution (10^–5^ M) at its natural pH and placed under direct natural sunlight with average solar radiation of 600 W/m^2^ (during the experiment.) without any stirring mechanism. Degradation test samples were collected at an interval of 30 min for 2 h. The absorption peaks were obtained by using the UV–vis spectrophotometer (Perkin Elmer). To examine the reusability test, the catalyst was collected via centrifugation and washed thoroughly with water and ethanol several times, then dried at 100 °C in a normal oven. Further, the study was extended to catalyst dosage variation, CIP concentration variation, and pH level variation of the solution. Catalyst dosage variation was performed by adding 50 mg, 100 mg, and 200 mg of ZnO NS to 100 ml 10^–5^ M of CIP solution. For variation of CIP concentration, 1 × 10^–5^ M, 2 × 10^–5^ M, and 5 × 10^–5^ M CIP solutions were deployed. For varying the pH level of the CIP solution, along with the natural pH, pH 3 and pH 10 solutions were prepared using a dedicated pH meter. In order to mimic real-world photodegradation, the sample was performed in natural sunlight without stirring and in a closed chamber to prevent evaporation and change of concentration.

### Biocompatibility assay using MTT

The cytotoxicity of synthesized ZnO NSs was monitored using the MTT assay. The MTT tetrazolium salt is reduced to formazan by the active enzymes present in metabolically active live cells and is directly proportional to the number of cells alive. A549 and A375 cells were cultured in 24-well plates with an initial cell density of 8 × 10^3^ cells per well for A549 cells and 9.3 × 10^4^ cells per well for A375 cells, respectively. DMEM supplemented with 10% FBS and 1% antibiotic (ampicillin) solution were provided to the cells for growth and incubated at 37 °C in a humidified atmosphere with a 5% CO_2_ level for 24 h. The A549 and A375 cells were treated with different concentrations of ZnO NSs (5 μg/ml 10 μg/ml, 50 μg/ml, 100 μg/ml), respectively and further incubated for 24 h. 5 mg/ml of MTT was added to each well and incubated under similar conditions 4 h in dark condition. After 4 h, 1 ml of DMSO to each well was added to dissolve the formazan crystals completely and the absorbance was noted at 570 nm using a UV spectrophotometer (Shimadzu UV-1800). The viable cell percentage was calculated using the following formula^70^.

$$\% \,{\text{cell}}\,{\text{viability}}=({\text{(O}}{\text{.D}}{.)}\,570\,{\text{of}}\,{\text{treated}}\,{\text{cells}}/{\text{(O}}{\text{.D}}{.)}\,570\,{\text{of}}\,{\text{untreated}}\,{\text{control}}\,{\text{cells}}) \times 100$$

### Live dead assay

According to the method described by Gowtham et al.^24^, we performed the Live dead assay. Acridine orange (AO) and ethidium bromide (EB) were used for detecting live cells and dead cells, respectively. The live cells produce green fluorescence and dead cells produce red/orange fluorescence under a fluorescent microscope. A549 and A375 cells were cultured on sterile coverslips which were placed inside a 35 mm tissue culture plate and maintained using the same condition as mentioned the experimental section. After 24 h, cell treatment was done with 10 μg/ml of ZnO and incubated for 24 h further. After incubation, the cells were treated with a mixture of two dyes (5 μl AO (1.2 mM)/2 μl EB (1.9 mM) dissolved in 2 ml of sterile PBS) under dark condition and incubated for 3 min at 37 °C in an incubator. After 3 min, the coverslips were positioned on a grease-free frosted sterile glass slide and the fluorescence image of live and dead cells was recorded under a fluorescent microscope (Olympus, BX 51) at 10X magnification.

### Antimicrobial activity of ZnO nanosheets

#### Minimum inhibitory concentration (MIC)

*E. coli* K12 and *S. aureus* were inoculated in 2 ml of freshly prepared Luria–Bertani (LB) broth followed by overnight incubation at 37 °C. After 24 h, the LB agar plates were prepared, and the inoculum of *E. coli* and *S. aureus* growing in the log phase were spread on the agar plates. The sterile discs of 6 mm diameter were positioned on each plate and ZnO was added at different concentrations on the discs (2.5, 5.0, 10, 50, 100 μg/ml). As a positive control, antibiotic antimycotic solution 100 X liquid was utilized. Overnight incubation was done at 37 °C followed by the measurement of the zone of inhibition around the discs. This experiment enables us to determine the MIC.

#### Minimum bactericidal concentration (MBC)

The antibacterial efficacy of ZnO was determined by using a standard broth dilution method. For this, the ZnO sample was serially diluted in concentrations (0.01, 0.1, 1, 10, 15 μg/ml for *E. coli* and 0.01, 0.1, 1, 10, 20 μg/ml for *S. aureus*). The doses were determined based on the MIC experiment results. A drop of subcultured fresh *E. coli* and *S. aureus* cultures was added and the control tube contained only culture-inoculated broth. Then the tubes were incubated at 37 °C for 24 h. After 24 h incubation, the samples were seeded in nutrient agar by spread plate technique and the plates were incubated at 37 °C for 24 h. Further, the colonies were counted. The bacterial killing at the minimum concentration of ZnO was determined by the MBC.

#### Scanning electron microscopy (SEM)

The visualization of the bacterial death mediated by the ZnO NSs, we conducted the SEM analysis. Four samples were analyzed, two untreated control bacteria (*E. coli* and *S. aureus*), and the other two samples of *E. coli* and *S. aureus* treated with 10 μg/ml of ZnO NSs and incubated for 24 h at 37 °C. The bacterial samples suspended in LB, were dropped on a grease free coverslip and air dried in a dirt free atmosphere. The SEM was captured using FEI Quanta FEG 200-High Resolution Scanning Electron Microscope (SEM) with appropriate magnification.

## Supplementary Information

Supplementary Information.

## Data Availability

Data will be made available on request to SS.
