# Supplementary material for: Photocatalytic degradation of antibiotics and antimicrobial and anticancer activities of two-dimensional ZnO nanosheets
Source: Sci Rep. 2024 May 6;14:10406. doi: 10.1038/s41598-024-59842-6 (PMC11579280; doi:10.1038/s41598-024-59842-6)
Supplement: Supplementary file 1 — Supplementary Information. [file 41598_2024_59842_MOESM1_ESM.pdf]

Photocatalytic Degradation of Antibiotics and Antimicrobial and Anticancer Activities of Two-Dimensional ZnO Nanosheets

Abhik Bhuin┼[a], Saranya Udayakumar┼[b], Janani Gopalarethinam,[b] Debdyuti Mukherjee,[c] Koyeli Girigoswami[b], Caroline Ponraj*[a], Sujoy Sarkar*[d,e]

[a] Physics Division, School of Advanced Sciences, Vellore Institute of Technology Chennai, Vandalur-Kelambakkam Road, Chennai-600127, Tamil Nadu, India

[b] Medical Bionanotechnology Laboratory, Faculty of Allied Health Sciences, Chettinad Hospital and Research Institute (CHRI), Chettinad Academy of Research and Education (CARE), Chettinad Health City, Kelambakkam, Chennai 603103, India

[c] Centre for Fuel Cell Technology (CFCT), International Advanced Research Centre for Powder Metallurgy and New Materials (ARCI), IIT-M Research Park, Taramani, Chennai 600113

[d] Chemistry Division, School of Advanced Sciences, Vellore Institute of Technology Chennai, Vandalur-Kelambakkam Road, Chennai-600127, Tamil Nadu, India

[e] Electric Vehicle Incubation, Testing and Research Centre (EVIT-RC), Vellore Institute of Technology Chennai, Vandalur-Kelambakkam Road, Chennai-600127, Tamil Nadu, India

e-mail: sujoy.sarkar@vit.ac.in

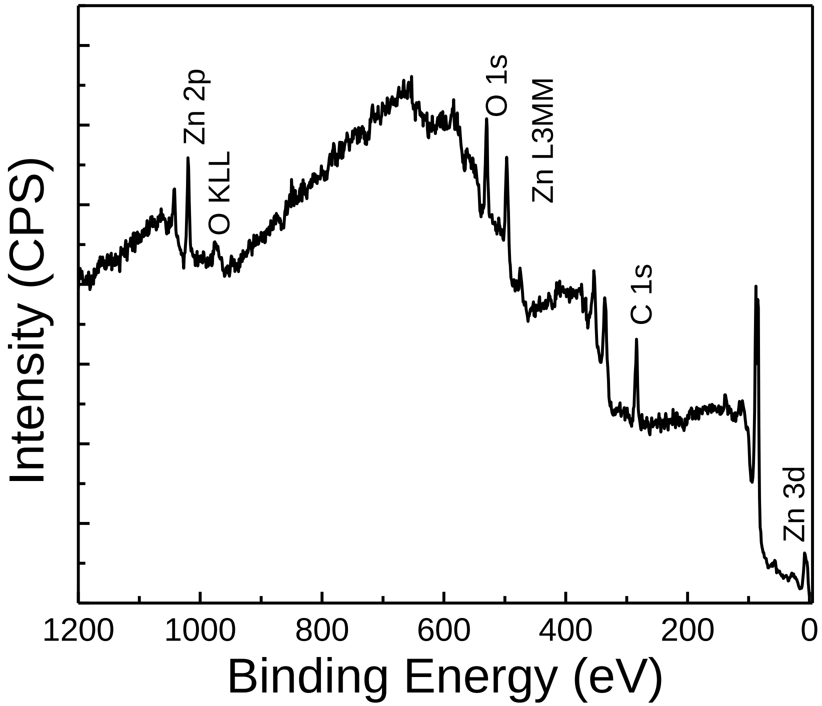

Fig. S1. XPS survey spectra of ZnO NSs.

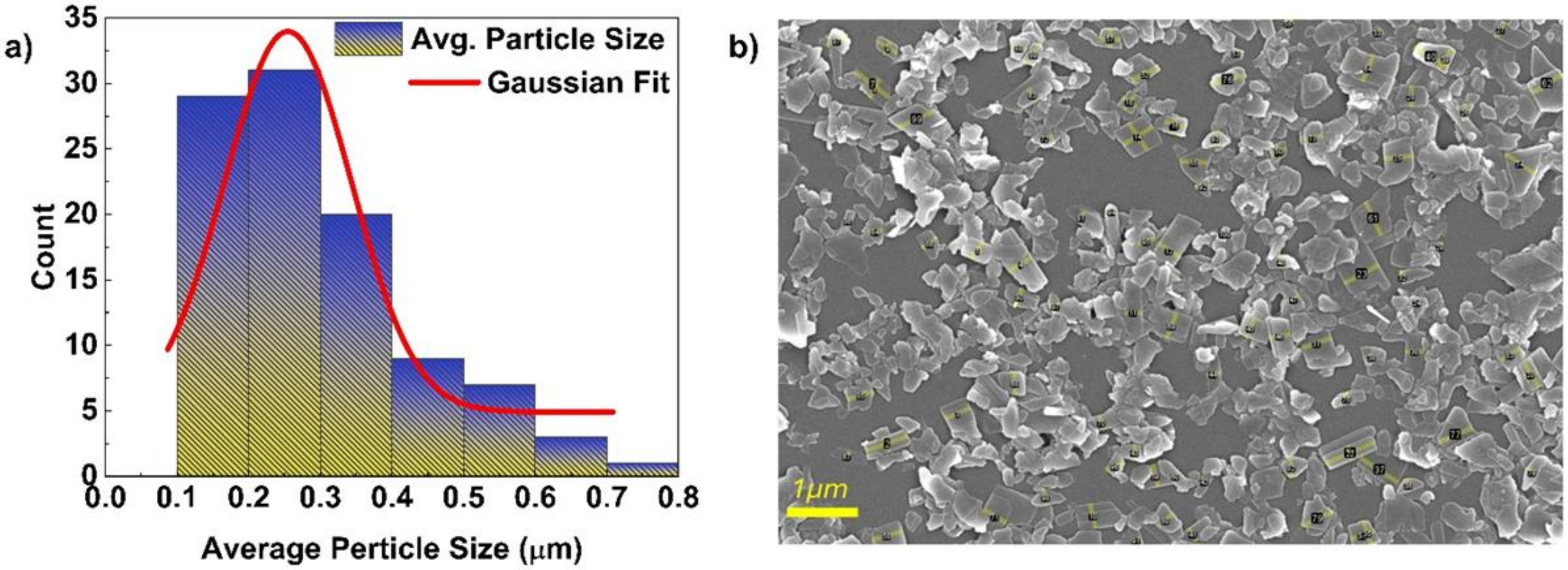

Fig. S2. Particle size distribution fitted by a Gaussian curve (a) and SEM (b) micrograph of ZnO NSs.

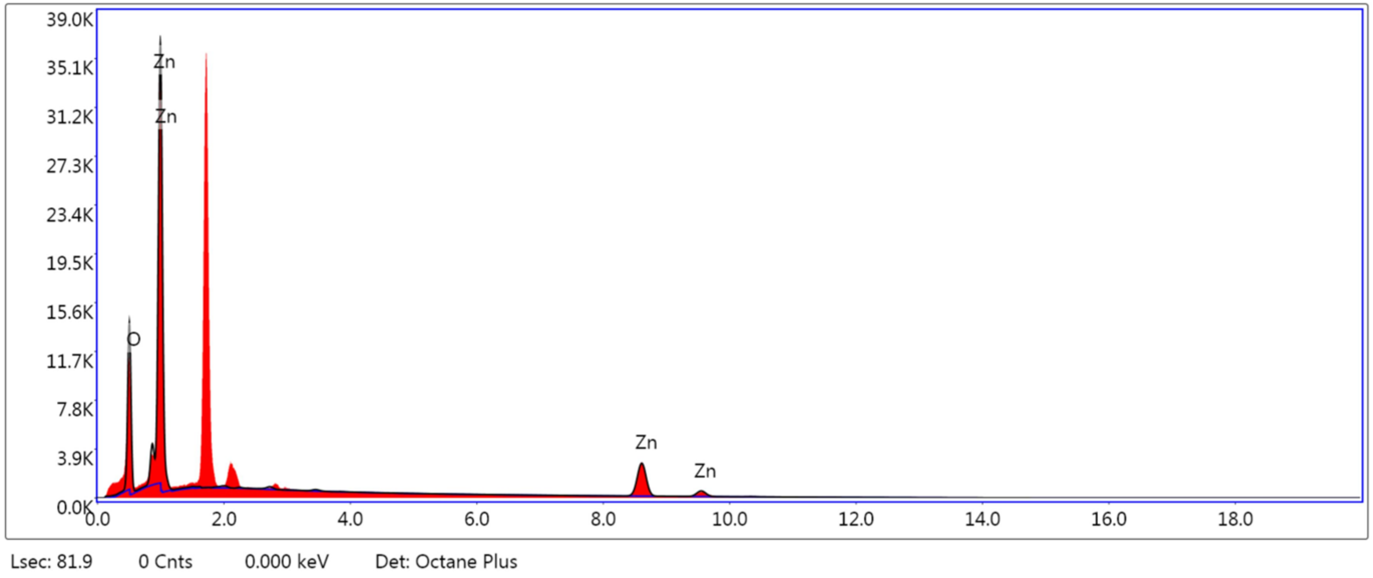

Element	Weight %	Atomic %	Net Int.	Error %	Kratio	Z	A	F
O K	22.16	53.77	2091.90	7.17	0.1191	1.2456	0.4315	1.0000
ZnK	77.84	46.24	1096.30	3.83	0.7135	0.9119	1.0022	1.0030

Fig. S3. EDS and atomic % of Zn and O present in ZnO NSs.

Photocatalytic Dye (MB) Degradation

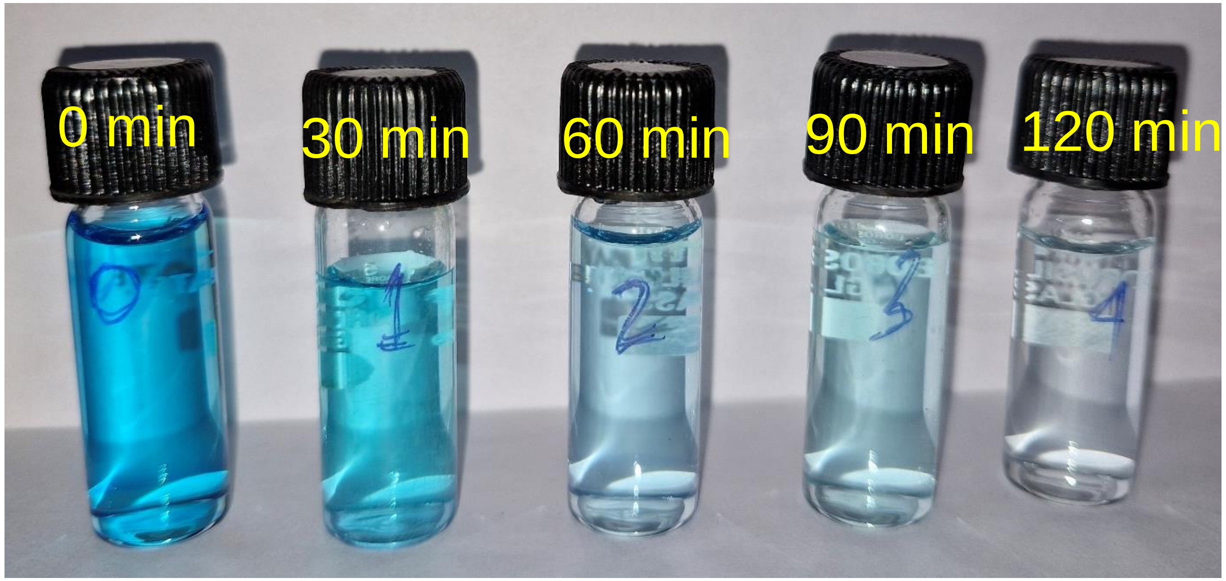

Fig. S4. Digital photographs of the MB (10^{-5} M) dye degradation at different irradiation time (0, 30, 60, 90, and 120 min).

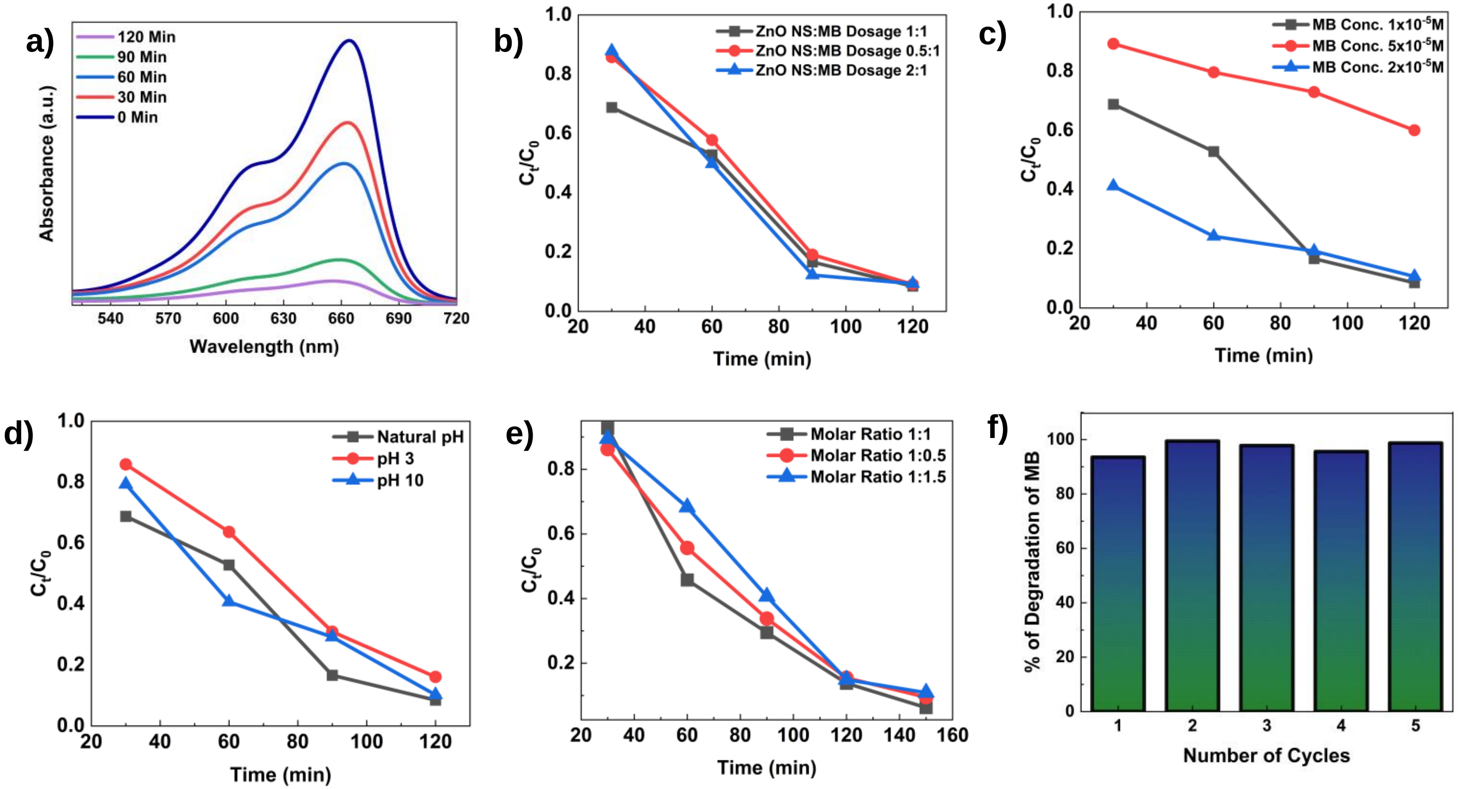

Fig. S5. a) Time-dependent UV−vis spectra of photocatalytic degradation of MB under direct sunlight using ZnO NSs. (b) Dosage Impact Study: Time-dependent photocatalytic degradation of MB under various dosage of ZnO NS (1:1, 1:0.5: 1:2). (c) Concentration Variation Analysis: Time-dependent photocatalytic degradation of MB by ZnO NS for different concentration of MB solution ($1x10^{-5}$M, $2x10^{-5}$M, $5x10^{-5}$M) (d) pH Level Influence: Time-dependent photocatalytic degradation of MB by ZnO NS for under different solution pH values (3, 7, and 10). (e) Precursor Molar Ratio Effect: Time-dependent photocatalytic degradation of

MB by ZnO NS prepared by varying the molar ratio (1:1, 1:0.5, 1:1.5) of the precursor. (f) Reusability Evaluation: Reusability study of ZnO NS in degradation of MB for 5 consecutive cycles

The trial run with MB provided the initial kinetics for photocatalytic degradation of ZnO NS. ZnO NS synthesized is 1:1 molar ratio shoed better photocatalytic activity and degradation kinetics compared to the 1:0.5 and 1:1.5 molar ratio. Catalyst dosage variation was performed by adding 25 mg, 50 mg, 100 mg of ZnO NS to 50 ml 10^{-5} M of MB solution. For variation of MB concentration $1x10^{-5}$ M, $2x10^{-5}$ M, $5x10^{-5}$ M MB solution was deployed and for pH level natural pH of the solution as well as pH 3, pH 10 solution. In order mimic real world scenarios degradation was performed in natural sunlight without stirring condition and in a closed chamber to prevent evaporation and change of concentration. At $2x10^{-5}$ M concentration we observed fastest degradation ≈60% degradation in first 30 min. Under the designed experiment condition, we could not significantly isolate influence of pH. In case MB we found the 1:1 dosage to provide sable kinetics for degradation. To further confirm we have studied the similar variation for CIP to further understand the degradation kinetics.

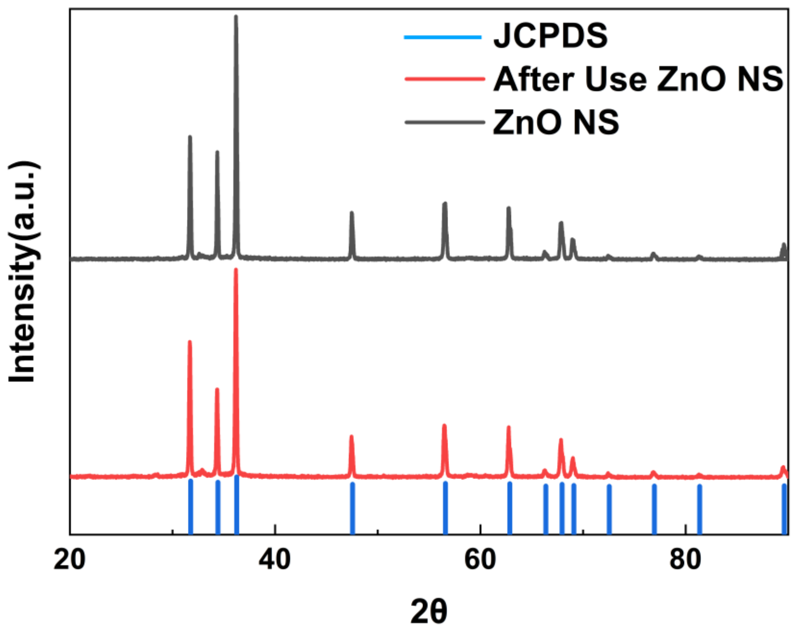

Fig. S6. Reusability study of ZnO NS in degradation of CIP for five consecutive cycles: Before and after usage XRD to confirm no loss in structural integrity of the synthesized material.

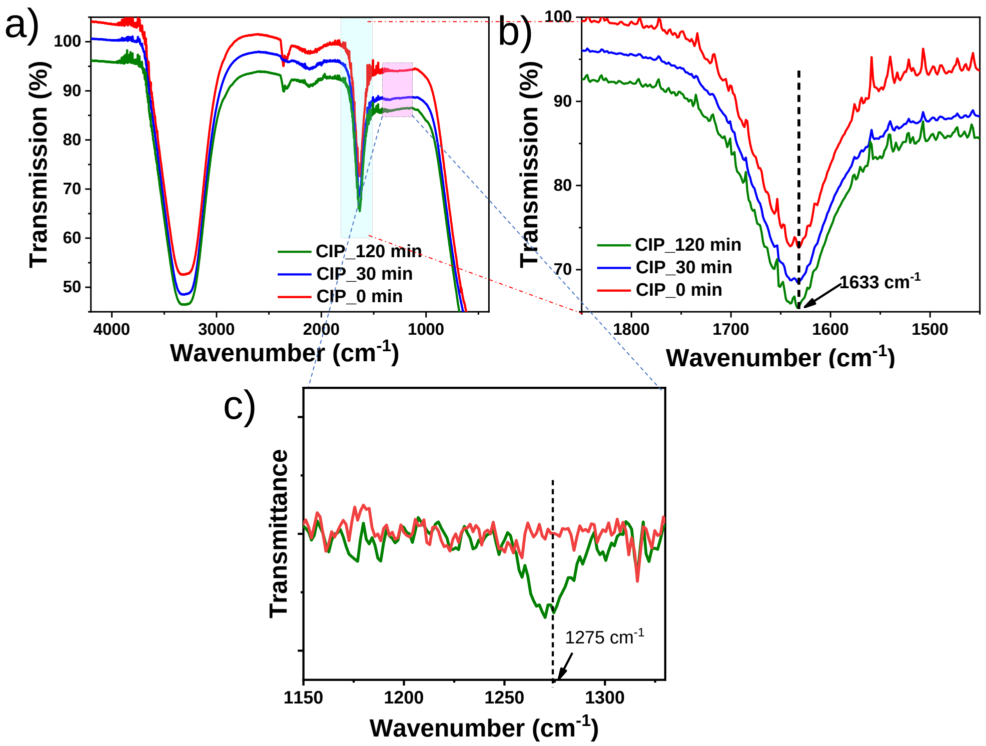

Fig S7. (a) IR analysis of CIP degradation at 0 min,30 min,120 min stage during the process of degradation (b) Zoomed portion (sky blue) of the Fig S7a. (c) Zoomed portion of the pink coloured box of the Fig S7a (baseline corrected).

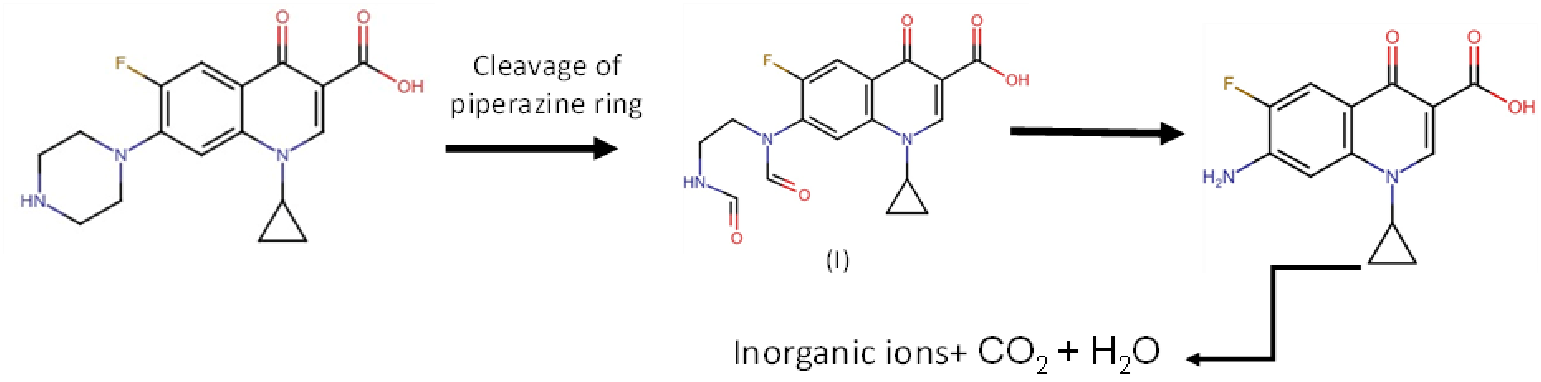

Scheme 2: Molecular mechanism of CIP degradation

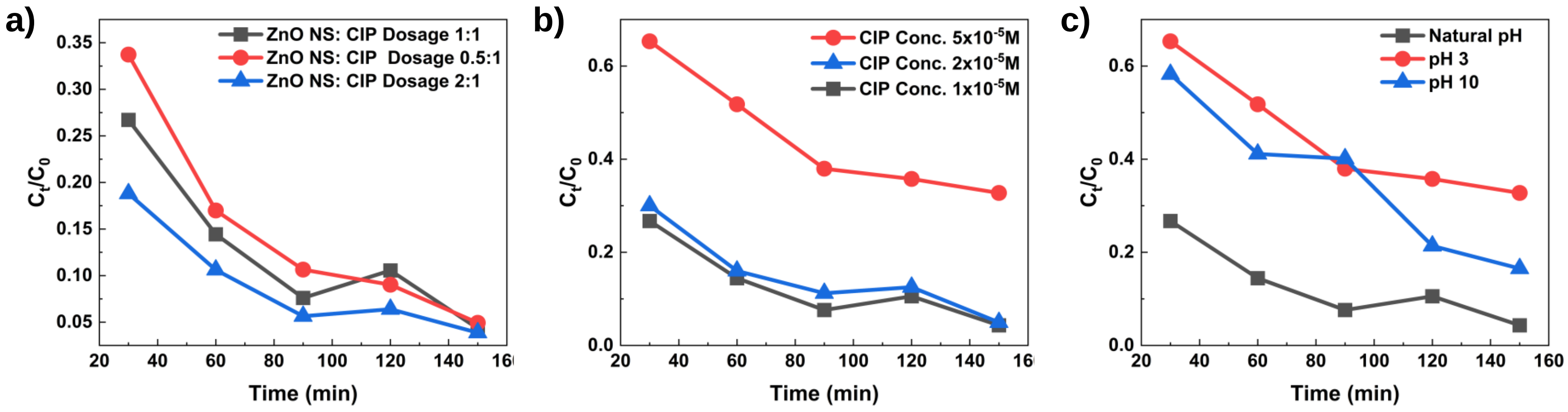

Fig. S8. (a) Dosage Impact Study: Time-dependent liquid-phase photocatalytic degradation of CIP under various dosage of ZnO NS to CIP Solution. (c) Concentration Variation Analysis: Time-dependent liquid-phase photocatalytic degradation of CIP by ZnO NS for different concentration of CIP solution (d) pH Level Influence: Time-dependent liquid-phase photocatalytic degradation of CIP by ZnO NS for under different solution pH values.

To understand the effects of catalyst dosage in CIP degradation, a different amount of photocatalyst was varied from 0.5 mg/ml to 2 mg/ml at natural pH and 10^{-5} M concentration and found that photodegradation of CIP is found to be effective at 0.5 mg/ml (Fig. S8a). The decrease in rate at higher dosage is attributed to a shielding effect from the excess catalyst, which hinders photocatalyst excitation due to photon

scattering. Additionally, higher catalyst concentrations combined with stirring less condition may lead to particle agglomeration and fewer available active sites, essential for photocatalysis.

To understand the effects of CIP concentration and limits of the catalyst in our designed experiment, a 1 mg/mL catalyst was added to 100 mL of 1×10^{-5} M, 2×10^{-5} M, 5×10^{-5} M CIP solutions (Fig. S8b). Similar degradation kinetics for 1×10^{-5} M and 2×10^{-5} M CIP solutions was observed, which might be the optimal concentration for CIP degradation for as synthesized ZnO NS in a similar environment. However, at higher concentration (5×10^{-5} M) of CIP, the photodegradation is found to be slower than other concentration. It is expected that in the presence of an optimal number of degradable molecules helps for faster kinetics to establish.

Since the pH of the CIP solution affects the chemical structure of the solution, pH is a crucial factor. To understand the effect of the pH of the solution, different pH (3, 7 and 10) of the solution were investigated (Fig S8c) and found that, the degradation kinetics was faster at neutral pH. It can be explained that at lower pH (<5.5), protonation at the $-NH_2$ functional groups causes CIP to exist in the cationic form. At pH values between 5.5 and 7.7, it takes the zwitterionic form, and at pH values higher than 7.7, it takes the anionic form. The ZnO NS photocatalyst exhibits a similar tendency to carry extra positive or negative charge that is depending on pH, which significantly lowers the degradation rate.

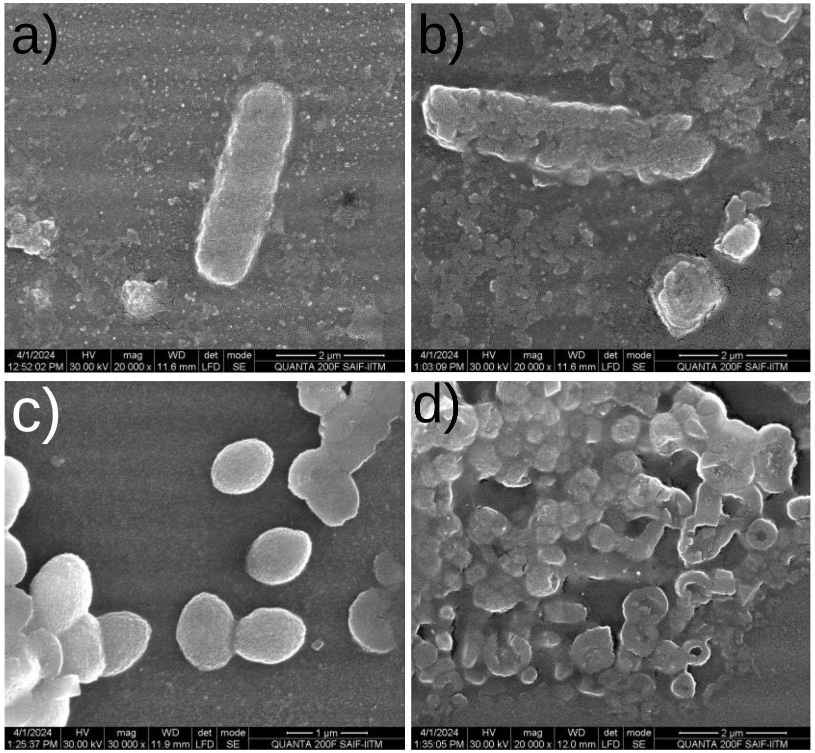

Fig. S9: The SEM images of (a) of *E. coli* (b) *E. coli* treated with 10 μg/ml of ZnO nanosheets, (c) *S. aureus* and (d) *S. aureus* treated with 10 μg/ml of ZnO nanosheets

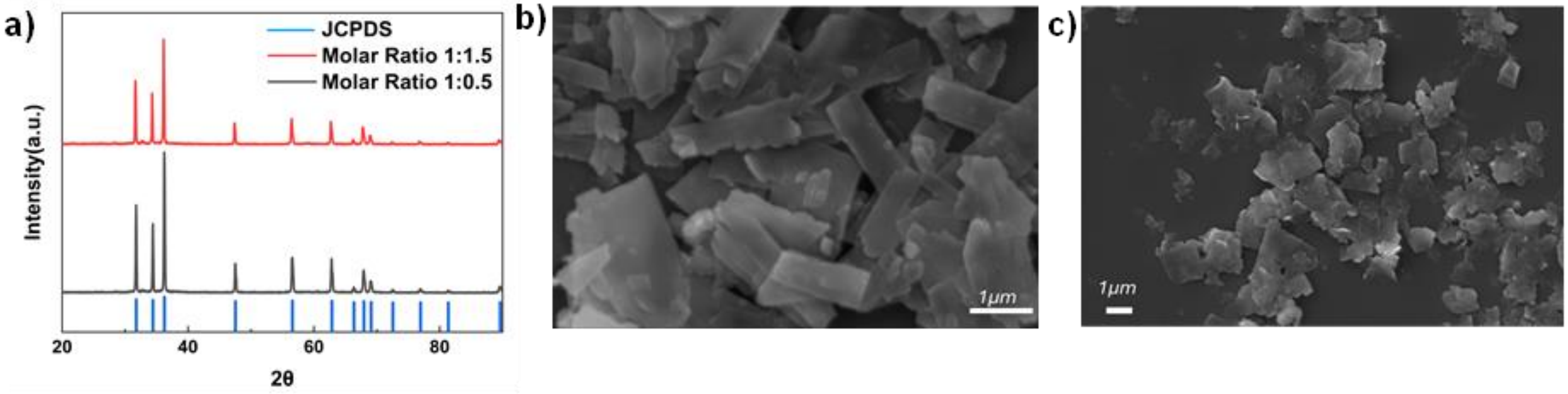

Fig. S10. (a) XRD analysis of as synthesized ZnO NS by varying precursors ratio against JCPDS. (b) FESEM Image of ZnO NS synthesized with precursor molar ratio 1:0.5 (c) FESEM Image of ZnO NS synthesized with precursor molar ratio 1:1.5

Supporting Table S1

Materials	DYE Degradation	Antimicrobial	Anticancer	Antibiotic Degradation	References
**ZnO NPs**	Name- Congo Red<br>Concentration Used- 10 ppm, 25 ml.<br>Dosage- 0.5mg<br>Efficiency- 95.9% at 25 min	Name- *Escherichia coli*<br>Concentration Used- 100 mg per ml.<br>Dosage-100 µL<br>Zone of Inhibition- 13±0.462 mm<br><br>Name- *Staphylococcus aureus*<br>Concentration Used- 100 mg per ml.<br>Dosage-100 µL<br>Zone of Inhibition- 10± 0.578 mm	-	-	[1]
**ZnO NSs**	Name- MB<br>Concentration Used- 10^{-5} M<br>Dosage- 1 mg/ml<br>Efficiency- Colourless	Name- *Escherichia coli*<br>Concentration Used- 100 µg/ml<br>Zone of Inhibition- 1.95±0.021 cm	Name- *A375*<br>Concentration Used- 100 mg per ml.<br>Time duration- 24 hr<br>%Cell Viability- 65.26±2.2	Name- CIP<br>Concentration Used- 10^{-5} M, 100 ml.<br>Dosage- 50 mg<br>Efficiency- ~90% at 120	**This Paper**

	after 150 min	Name- *Staphylococcus aureus*<br>Concentration Used- 100 µg/ml<br>Zone of Inhibition- 1.95± 0.007 cm	Name- *A549*<br>Concentration Used- 100 mg per ml.<br>Time duration- 24 hr<br>%Cell Viability- 65.82±4.10	min	
**ZnO NP**	-	-	Name- *A549*<br>Concentration Used- 125 mg per ml.<br>Time duration- 72Hr<br>%Cell Viability ≈ 25	-	[2]
**Commercial ZnO**	-	-	Name- *A549*<br>Concentration Used- 125 mg per ml.<br>Time duration- 72Hr<br>%Cell Viability ≈ 65	-	[2]
**Nanocrystalline ZnO**		Name- *Escherichia coli*<br>Concentration Used- 0.1 mg per ml.<br>Duration- 3Hr<br>Maximum % reduction in Viability- 99.8<br>Name- *Staphylococcus aureus*<br>Concentration Used- 0.1 mg per ml.<br>Duration- 3Hr<br>Maximum % reduction in Viability- 98			[3]

**ZnO NPs**	-	-	Name- *A549*<br>Dosage- 70µg<br>Time duration- 24Hr<br>%Cell Viability ≈ 5	-	[4]
**ZnO nano pellets**	-	Name- *Escherichia coli*<br>Dosage- 62.5 µg<br>Zone of Inhibition- 12±1 mm<br>Name- *Staphylococcus aureus*<br>Dosage- 62.5 µg<br>Zone of Inhibition- 18.75± 0.125 mm	Name- *A549*<br>Concentration/ Dosage- 300 µg/mL<br>Time duration- 24Hr<br>%Cell Viability ≈ 75	-	[5]
**ZnO NP**	Name- MB<br>Concentration Used- 0.5 mM,<br>Dosage- 20 mg<br>Efficiency- 29% after 30 min	Name- *Staphylococcus aureus*<br>Concentration Used- 0.1 mM<br>Maximum % reduction in Viability- >80	-	-	[6]
**ZnO**	-	Name- *E. coli*<br>Concentration Used- 125 µg/mL.<br>Maximum % reduction in Viability ≈ 60<br>Name- *Staphylococcus aureus*<br>Concentration Used- 1000ppm.<br>Maximum % reduction in Viability ≈ 75	Name- *A549*<br>Concentration/ Dosage-125 µg/mL<br>Time duration- 24Hr<br>%Cell Viability ≈ 25	-	[7]

**ZnO**	-	Name- *Escherichia coli*<br>Dosage- 1000 ppm<br>Zone of Inhibition- 1.94 ± 0.04 mm	-	-	[8]
		Name- *Staphylococcus aureus*<br>Dosage- 62.5 µg<br>Zone of Inhibition- 1.90 ± 0.13 mm			

References:

1. Verma, R., et al., *Green synthesis of ZnO NPs using Timur (Zanthoxylum armatum DC.) plant extract for antimicrobial and dye degradation applications.* Chemical Papers, 2023. **77**(9): p. 5587-5597.
2. D, S., et al., *Anti cancer activity of zno nanoparticles on MCF7 (breast cancer cell) and A549 (lung cancer cell).* ARPN Journal of Engineering and Applied Sciences, 2015. **10**: p. 5418-5421.
3. Applerot, G., et al., *Enhanced Antibacterial Activity of Nanocrystalline ZnO Due to Increased ROS-Mediated Cell Injury.* Advanced Functional Materials, 2009. **19**(6): p. 842-852.
4. Parthasarathy, R., et al., *Zinc Oxide Nanoparticles Synthesized by Bacillus cereus PMSS-1 Induces Oxidative Stress-Mediated Apoptosis via Modulating Apoptotic Proteins in Human Melanoma A375 Cells.* Journal of Cluster Science, 2022. **33**(1): p. 17-28.
5. Gopala Krishna, P., et al., *ZnO nanopellets have selective anticancer activity.* Materials Science and Engineering: C, 2016. **62**: p. 919-926.
6. Joe, A., et al., *Antimicrobial activity of ZnO nanoplates and its Ag nanocomposites: Insight into an ROS-mediated antibacterial mechanism under UV light.* Journal of Solid State Chemistry, 2018. **267**: p. 124-133.
7. Mohamad Sukri, S.N.A., et al., *Enhanced antibacterial and anticancer activities of plant extract mediated green synthesized zinc oxide-silver nanoparticles.* Frontiers in Microbiology, 2023. **14**.
8. Droepenu, E.K., et al., *Biosynthesis, characterization, and antibacterial activity of ZnO nanoaggregates using aqueous extract from Anacardium occidentale leaf: comparative study of different precursors.* Beni-Suef University Journal of Basic and Applied Sciences, 2021. **10**(1): p. 1.
